# The renal response to FGF23 shifts from phosphaturia toward inflammation in kidney disease

**DOI:** 10.1002/ccs3.70061

**Published:** 2026-02-23

**Authors:** Matthias B. Moor, Mikhail Burmakin, Anna Levin, Gül Gizem Korkut, David Brodin, Annika Wernerson, Annette Bruchfeld, Peter Bárány, Anna Witasp, Jaakko Patrakka, Hannes Olauson

**Affiliations:** ^1^ Division of Renal Medicine Department of Clinical Science Intervention and Technology Karolinska Institutet Stockholm Sweden; ^2^ Division of Pathology Department of Laboratory Medicine Karolinska Institutet Stockholm Sweden; ^3^ Department of Nephrology and Hypertension University Hospital Bern and University of Bern Bern Switzerland; ^4^ Bioinformatics and Expression Analysis Core Facility (BEA) Karolinska Institutet Huddinge Sweden; ^5^ Department of Health Medicine and Caring Sciences Linköping University Linköping Sweden

**Keywords:** cytokines, FGF23, inflammation, kidney disease, proteinuria, RNA deconvolution

## Abstract

FGF23 excess is associated with morbidity and mortality, but the role of excessive circulating FGF23 concentrations as a causative factor of pathology is controversial. Here, we investigated the consequences of FGF23 excess in kidney disease. This study used three disease models: anti‐glomerular basement membrane (anti‐GBM) disease, Adriamycin nephropathy, and adenine‐containing diets. Anti‐GBM and Adriamycin mice and matched control mice received recombinant FGF23 (1 µg) or vehicle for six days (anti‐GBM) or once (Adriamycin model), with dissection 24 h after the last injection. We established precision‐cut kidney slices (PCKS) in adenine nephropathy for 24 h of treatment with recombinant FGF23 or vehicle. We assessed serum cytokines, biochemistry, and renal transcriptomes and histology of mice and patients with IgA nephropathy. RNA‐Seq data and published transcriptomes underwent gene set enrichment, bulk ligand–receptor interaction analysis, and cell‐type decomposition. Experimental anti‐GBM disease caused decreased glomerular filtration rate, albuminuria, and renal tubular casts. FGF23 treatment increased phosphaturia and circulating soluble tumor necrosis factor receptor 1. The anti‐GBM model showed FGF23‐driven proinflammatory transcriptional signatures and Vcam1, Pdgfrb, and chemokine signaling, which were absent in FGF23‐treated healthy mice. FGF23 increased renal macrophage content by transcriptome deconvolution and by immunofluorescence. In Adriamycin‐induced nephropathy and in PCKS from adenine nephropathy, short‐term FGF23 excess increased proinflammatory transcripts. Human data revealed associations between circulating FGF23 and renal immune cell infiltration. We found FGF23‐driven renal patterns of proinflammatory gene and protein expression or leukocyte overabundance. The present data provide evidence that excess FGF23 directly drives inflammation in kidney disease and may serve as a therapeutic target.

## Graphical abstract

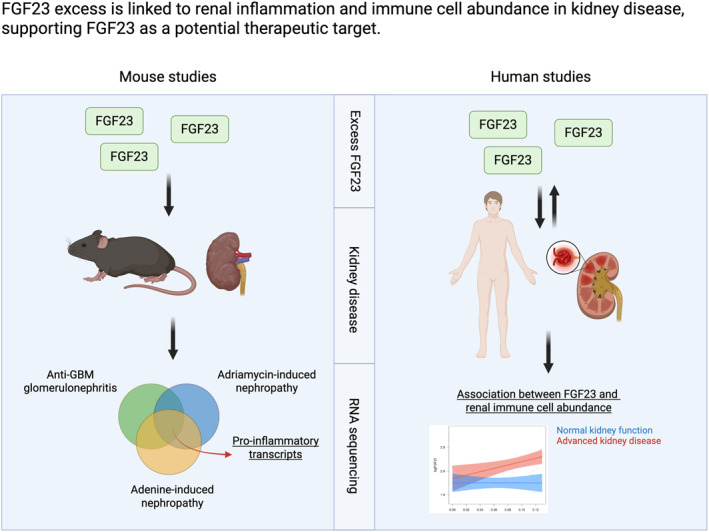

## 1 INTRODUCTION

Patients with chronic kidney disease (CKD) experience substantial morbidity due to earlier and more frequently occurring cardiovascular events compared to the general population, in part driven by uremic toxins, a systemic inflammatory state, endothelial dysfunction, and vascular calcification. Vascular and other soft‐tissue calcification is, in part, caused by the failure of the kidney to sufficiently excrete phosphate due to an end‐organ resistance of the diseased kidney to the bone hormone fibroblast growth factor 23 (FGF23).^1^ Circulating concentrations of FGF23 can rise up to 1000‐fold in CKD, causing potential off‐target effects and alterations in cellular signaling.^2^ Experimental studies in mice showed that FGF23 excess drives left heart hypertrophy via FGF receptor 4 (FGFR4), calcineurin, and nuclear factor of activated T cells (NFAT).^3^, ^4^, ^5^ Intriguingly, adverse effects of FGF23 excess on the heart are independent of systemic phosphate load, and FGF23 acts synergistically with phosphate toxicity.^6^, ^7^ In addition, FGF23 also causes hepatocytes to produce acute‐phase proteins in a calcineurin‐dependent manner that may drive proinflammatory actions in other organs.^8^ However, renal adverse effects of FGF23 in CKD are less understood and, if they exist, underlie other mechanisms: Absence of FGFR4, the key mediator of cardiac adverse effects of FGF23, did not alter experimental CKD progression.^9^ Further, studies on adverse renal effects of FGF23 in mice revealed conflicting results: Others and we previously reported that systemic *Fgf23* overexpression decreased renal expression of *Klotho*, a key co‐receptor for FGF23 required for normal renal function.^10^, ^11^ Exogenic delivery of FGF23 also induced kidney fibrosis and profibrotic cellular signaling in injury‐primed renal fibroblasts.^11^, ^12^ In contrast, completely binding FGF23 by antibody in rats increased CKD mortality.^13^ Further, *Fgf23* gene delivery had protective effects on endothelium and reduced AKI severity in mice with renal ischemia‐reperfusion injury and contralateral nephrectomy.^14^

Among human studies, most observational data showed associations between circulating FGF23 and adverse cardiovascular outcomes, mortality, and also adverse renal outcomes in several consortia.^3^, ^15^, ^16^, ^17^ However, when assessing a potential causal effect by Mendelian randomization among the general population, genetically predicted circulating FGF23 concentrations were not associated with renal function.^18^ Even more, a meta‐analysis concluded that FGF23 is likely not causative for cardiovascular complications because of an absent exposure–response relationship and because noncardiovascular complications are also associated with FGF23 concentrations similar to those of cardiovascular complications.^15^

In the light of these controversies, it remains unresolved in the field whether FGF23 is merely a biomarker or a real causative agent of morbidity, such as CKD progression. With the intent of reducing this gap in knowledge, we performed experimental and observational studies on the adverse renal effects of FGF23 excess with a focus on CKD pathophysiology. Based on experimental glomerular disease with proteinuria‐induced tubular injury, data from genetically induced FGF23 excess in mice, and biochemical and advanced transcriptome analyses in patients with CKD, we, here, report that FGF23 induces proinflammatory cytokine expression and leukocyte migration to kidney tissue in kidney disease. Further, in isolated precision‐cut kidney slices (PCKS), we discovered kidney‐intrinsic cytokine expression in response to FGF23 in murine CKD, thus providing evidence of liver‐independent proinflammatory effects of FGF23 in kidney disease.

## 2 MATERIALS AND METHODS

### 2.1 Sex as a biological variable

Sex was not considered as a biological variable, and similar findings are expected for both sexes. Animals and human patients of both sexes were included, as reported in the respective sections.

### 2.2 Animal experiments

Mice of the C57BL/6J, DBA/2J, and BALB/c strains were purchased from Charles River or Scanbur. Mice were housed in single‐ventilated cages with a 12‐h light–12‐h dark cycle and had ad *libitum* access to water and standard chow (Lantmannen) unless indicated otherwise. Temperature was constant at 20 ± 2°C, and relative humidity was 50 ± 5%. In all mouse experiments, euthanasia was performed by terminal exsanguination and cervical dislocation under anesthesia with isoflurane USP (Baxter), followed by organ collection.

### 2.3 Kidney disease models

We performed animal experiments using 3 mouse models of kidney disease summarized in Supporting Information Table S1 and listed below:

i) Anti‐glomerular basement membrane (anti‐GBM) disease was induced in C57BL/6 mice, as described earlier.^19^ In brief, male mice aged 8 weeks were subcutaneously immunized with 400 μL/kg of sheep IgG in Freund's complete adjuvant (F5881, Sigma‐Aldrich). After four days, the mice were intravenously injected with 130 μL of nephrotoxic serum (NTS) obtained from Probetex (PTX‐001S). Control mice were treated with vehicle (PBS in the corresponding volume).
ii) For induction of an Adriamycin nephropathy resembling human focal segmental glomerulosclerosis (FSGS), we chose to extend the investigation to the female sex: Female BALB/c mice aged 8 weeks received a single intravenous injection of 10.5 mg/kg body weight Adriamycin (Sigma) that was diluted to 2 mg/mL in 0.9% NaCl_2_ or vehicle (0.9% NaCl_2_ in the corresponding volume), derived from a protocol established earlier in the laboratory.^20^
iii) A model of toxic tubulointerstitial kidney disease was established in male DBA/2J mice using adenine, as described earlier.^21^ In brief, male DBA/2J mice aged 8 weeks were fed a casein‐based diet containing 0.2% of adenine (ssniff Spezialdiäten GmbH). Throughout the experiments, weight, and overall appearance of all mice were scored 3 times per week. Spot urine and serum were collected at several time points during the study.

### 2.4 Hormone injections

Treatments with FGF23 hormone were applied using a modification of an earlier protocol.^22^ Recombinant human FGF23 was obtained from R&D Systems, Minneapolis, MN, USA, and distributed via Thermo Fisher (Cat. #100‐52). Mice of the anti‐GBM model and controls were randomly allocated to receive daily intravenous injections of 1 μg of FGF23 suspended in PBS‐BSA 0.1% (Sigma) or PBSA‐BSA 0.1% vehicle starting 1 day after receiving NTS. Mice of the Adriamycin model and corresponding controls were randomized for a single intravenous injection of 1 μg of FGF23 or PBS‐BSA 0.1% vehicle. Mice of both disease models were euthanized 24 h after the last injection of FGF23.

### 2.5 Glomerular filtration rate (GFR) assessment

To assess GFR in the anti‐GBM model at baseline and 7 days after disease induction, a transdermal fluorescence detector (MediBeacon) was placed on the shaved back and was used to measure and quantify the clearance of injected FITC‐sinistrin, as previously described.^23^

### 2.6 Precision‐cut kidney slices (PCKS)

PCKS were established from DBA/2J mice with adenine nephropathy using a protocol modified from a previous study of the laboratory.^24^ No disease‐free controls were used for PCKS. In brief, a Compresstome® VF‐310‐0Z Vibrating Microtome (Precisionary Instruments) filled with William’s E Medium GlutaMAX‐I buffer (WME solution, Thermo Fisher) containing 25 mM D‐glucose (Merck) was used to obtain 300 µm kidney slices containing approximately 2/3 cortex and 1/3 medulla. The slices were transferred to 12‐well plates and put into 1.5 mL of WME solution at 37°C and 95% oxygen for 20 h. Treatments were performed with PBS‐BSA 0.1% vehicle or recombinant human FGF23 (R&D Systems, #100‐52) at 200 ng/mL, followed by harvesting and freezing in 1 mL of RNAlater solution (Sigma‐Aldrich), direct storage at −80°C, or fixing overnight in tubes containing 4% formaldehyde (Sigma‐Aldrich).

### 2.7 Serum and urine biochemistry assessment

Biochemical parameters were analyzed using quantification kits according to the manufacturer's instructions, including blood urea nitrogen (BUN) using the BUN Colorimetric Detection Kit (EIABUN, Thermo Fisher), creatinine by colorimetric detection using a kit (K625, BioVision), and phosphate by a colorimetric kit (Cat. MAK‐030, Merck). Murine FGF23 was detected using ELISA kits for intact FGF23 (Immutopics, Inc., Cat. #60–6800) and C‐terminal FGF23 (Immutopics, Inc., Cat. #60–6300), and human FGF23 was measured using an ELISA kit for intact FGF23 (Immutopics, Inc., Cat. #60–6600), as per the manufacturer's instructions.

### 2.8 Immune protein array

For quantification of 40 inflammation‐related proteins in mouse serum, two Mouse Inflammation Array C1 kits (RayBiotech, #AAM‐INF‐1‐8) were used as per the manufacturer's instructions, with 60 µL of serum from up to three mice pooled per sample, for a total of 4 experimental groups with 4 independent biological replicates per group. Membranes were bathed in the kits' chemiluminescence reagents for 30 s. Images of the membranes were obtained on an Odyssey® XF Imaging System (LICORbio). Spots on digital images underwent densitometric quantitation using an ImageJ Protein Array Analyzer macro,^25^ with standard settings and linear background subtraction. The obtained data were analyzed using R/limma with Benjamini–Hochberg correction for multiple testing and interaction analysis in a two‐factorial design (factors: treatment with FGF23 vs. vehicle and disease state: anti‐GBM vs. healthy).

### 2.9 Histology

Organs were fixed in 4% formaldehyde and underwent paraffin embedding. Four‐μm‐thick sections were made, followed by standard staining using hematoxylin and eosin or the Masson's Trichrome Stain Kit (Cat. #HT15, Sigma‐Aldrich). Brightfield images were scanned using a ZEISS Axioscan 7 scanner. The presence or absence of renal tubular protein casts was determined by the following score: 0, no casts; 1, >0–20%; 2%, 20%–40%; 3%, 40%–60%; 4%, 60%–80%; 5, >80% of the tubules are affected.

### 2.10 Immunofluorescence

Paraffin‐embedded sections were dewaxed and rehydrated, followed by antigen retrieval at 98°C for 20 min in 10 mM Tris, 1 mM EDTA, and 5% Tween. Subsequently, slides were washed with PBS before blocking with Dako serum‐free protein block (X0909, Agilent) for an hour at room temperature. After this, slides were incubated overnight at 4°C with primary antibodies against CD45 (Abcam ab61100, 1:100) or F4/80 (Invitrogen, Cat. #14‐4801‐82, 1:50). Alexa Fluor‐conjugated secondary antibodies were from Invitrogen. Slides were co‐stained with Hoechst 33,342 (H3570, 1:10,000, Life Technologies) for 10 min at room temperature. Sudan Black B (0.1% in 70% ethanol, Cat. #199664, Sigma‐Aldrich) was applied for 25 min at room temperature, followed by mounting in Dako fluorescent medium (S3023, Agilent). Slides were scanned at 20X magnification using a Vectra 3 automated quantitative pathology imaging system (Akoya Biosciences). Subtraction of background signals and image export were performed using inForm® software (Akoya Biosciences). Images were exported for quantification of stained cells using QuPath version 0.5. After automated cell detection, the fraction of CD45‐positive immune cells underwent automated quantification using an intensity threshold, and F4/80‐positive macrophages underwent manual identification by a blinded investigator while comparing with the autofluorescence channel. For CD45 staining, the fraction of positive cells out of approximately 7000 cells per animal was reported, and for F4/80, approximately 3000 cells per animal were analyzed.

### 2.11 RNA isolation

A fragment of the renal pars intermedia per mouse was homogenized and lysed using a TissueLyser (QIAGEN) in 600 μL RLT buffer + DTT. RNA was then extracted using QIAGEN RNeasy Mini Kit (QIAGEN) according to the manufacturer's instructions.

### 2.12 RNA‐Seq (Illumina mRNA ligation kit)

Total RNA was subjected to quality control with the Agilent TapeStation according to the manufacturer's instructions. To construct libraries suitable for Illumina sequencing, the Illumina TruSeq Stranded mRNA ligation is used. A sample preparation protocol including cDNA synthesis, ligation of adapters, and amplification of indexed libraries was used. The yield and quality of the amplified libraries were analyzed using Qubit by Thermo Fisher and the Agilent TapeStation. The indexed cDNA libraries were normalized and combined. The pools were sequenced on the Illumina NextSeq 2000 with a P3 100 sequencing run generating 58 bp paired‐end reads.

### 2.13 Bulk RNA‐Seq data analysis

Demultiplexing was performed using bcl2fastq (v2.20.0). Reads were aligned to GRCm38 from Ensembl using STAR (v2.7.9a). Aligned reads were assigned to coding regions of various biotypes defined in Mus_musculus.GRCm38.102.gtf from Ensembl using featureCounts (v1.5.1). Annotations were collected from Ensembl BioMart using the biomaRt package. All comparisons were performed using the DESeq2 package. For anti‐GBM and Adriamycin experiments, the 3 contrasts, namely, disease status (kidney disease model vs. healthy controls), treatment status (FGF23 vs. vehicle), and the interaction between disease status and treatment status, were tested, with false discovery rates adjusted for the 3 contrasts combined by the Benjamini–Hochberg method. Gene set enrichment analysis (GSEA) was performed using the fgsea package with the Molecular Signatures Database (MSigDB) collection of gene sets. Ligand–receptor interaction analysis was performed using BulkSignalR v.0.0.9^26^ in differential mode.

### 2.14 Transcriptome dataset retrieval from public repositories

Publicly available microarray datasets of renal gene expression were retrieved from the Gene Expression Omnibus dataset browser, including kidney dataset GDS3361 of 8‐week‐old male *Fgf23* transgenic and control mice by Marsell et al.^27^ and kidney dataset GDS879 of 5‐week‐old sex‐balanced (60% males) mice carrying the Hyp mutation in the *Phex* gene (Supporting Information Table S1).^28^ Single‐cell RNA‐Seq data were downloaded from the Gene Expression Omnibus, including GSE107585 of murine kidney from 7 sex‐mixed healthy C57BL/6 mice by Park et al. and GSE199711 of human kidney from 3 patients with kidney disease and 2 nondiseased nephrectomy specimens by Lake et al.^29^, ^30^

### 2.15 Human cohort of patients with IgA nephropathy

We reanalyzed published data from 59 adult patients with IgA nephropathy (IgAN) from the Karolinska Kidney Biopsy Project in relation to demographic and clinical characteristics, medication data, and serum biochemistry.^31^ In addition, the diagnostic kidney biopsy evaluations were accessed, including the Oxford classification, and we obtained the processed RNA‐Seq count data from microdissected nonglomerular (tubular) fractions of the kidney. The RNA‐Seq analysis procedure, including filtering and normalization, is described in Levin et al.^31^

### 2.16 Bulk transcriptome deconvolution

For analyses of relative renal segment or cell‐type abundance, single‐cell RNA‐Seq datasets GSE107585 and GSE199711 were reanalyzed using Seurat 5.0.3. After excluding cells with less than 200 or more than 2500 RNA counts or cells with >30% mitochondrial RNA, normalization and clustering were performed, and clusters were manually annotated based on cluster‐specific expression and markers with reference to the original publications of the datasets.^29^, ^30^ Subsequently, Bisque v.1.0.5^32^ was used in reference‐based decomposition mode for deconvolution of bulk RNA‐Seq or microarray datasets.

### 2.17 Statistics

Data analyses and visualizations were made with RStudio 2023.12.1 and/or GraphPad Prism 8 or SuperPlotsOfData.^33^ Frequency data were analyzed by the chi‐square test, and continuous data were analyzed by the *t*‐test, paired *t*‐test, or two‐way ANOVA, as appropriate. Univariable and multivariable linear regression models were used with dependent variables as indicated, using adjustments for immune‐relevant confounders for multivariable models, namely, 25OH‐vitamin D, PTH, corticosteroid use (yes/no), and other immunosuppressants (yes/no). In the models, we also included an interaction term of measured GFR and cell abundance. Circulating immune proteins and RNA‐Seq data were analyzed in a 2‐factorial design using limma and DESeq2, respectively, as reported above.

### 2.18 Study approval

All animal experiments were carried out at Karolinska Institutet following the protocols approved by the Ethical Committee on Research Animal Care (Linköping Animal Experimentation Ethics Committee; DNR 1336). The Karolinska Kidney Biopsy Project was approved by the Swedish Ethical Review Authority. The reanalysis of existing transcriptomes in the public domain required no authorization.

## 3 RESULTS

### 3.1 Partial renal resistance of the phosphaturic response to FGF23 in mice with anti‐GBM disease

**FIGURE 1:**
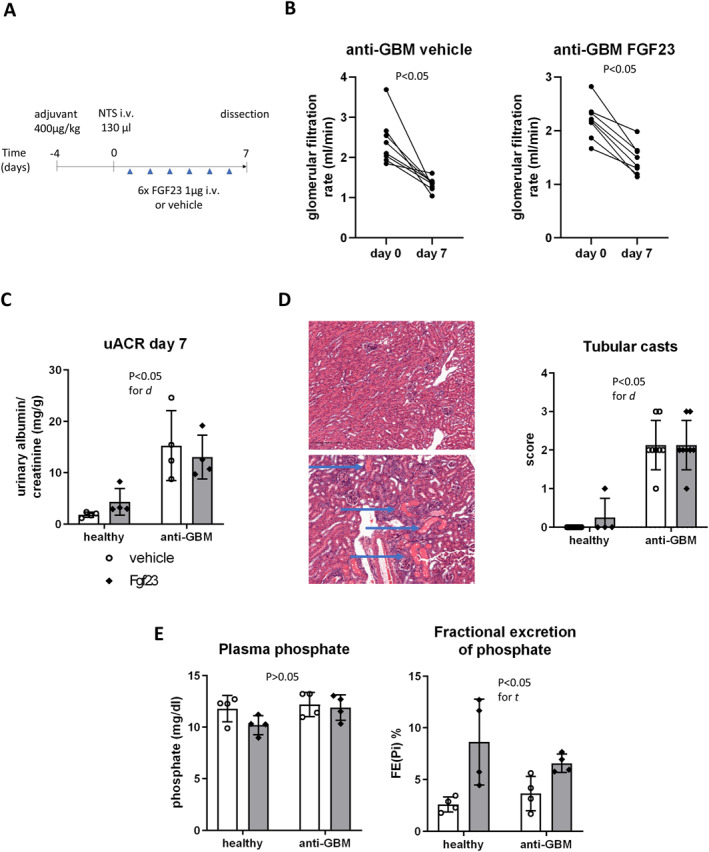
Anti‐GBM disease causes tubular damage and partial renal resistance to FGF23. (A) Depicts the experimental workflow in male C57BL/6 mice undergoing induction of anti‐GBM disease using nephrotoxic serum followed by 6 days of intravenous (IV) FGF23 or vehicle treatment. (B) Shows the glomerular filtration rate of different experimental groups at days 0 and 7. (C) Shows urinary albumin/creatinine ratio at day 7. (D) Shows the example renal sections negative or positive for renal tubular casts (arrows) and quantitative tubular cast scores. (E) Shows plasma phosphate and fractional excretion of phosphate of healthy mice and mice with anti‐GBM treated with vehicle or FGF23. Analyses in panel (B): paired *t*‐test. Analyses in panels (C–E): two‐way ANOVA. Anti‐GBM, anti‐glomerular basement membrane; d, disease state; t, treatment.

Previous work by others and us has shown systemic proinflammatory responses occurring under FGF23 excess in mice.^8^, ^10^ In this study, we aimed to substantiate the renal consequences of such adverse effects of FGF23 excess, using 2 mouse models with proteinuria‐induced proximal renal tubular dysfunction. We first induced anti‐GBM disease in adjuvant‐immunized C57BL/6 mice using nephrotoxic serum (Figure 1A), as previously described.^19^ Mice additionally received daily intravenous administrations of recombinant FGF23 or vehicle over 6 days (Figure 1A). We have previously assessed the morphological effects of this protocol of anti‐GBM disease induction using electron microscopy of the kidney, in which we detected severe podocyte effacement.^19^ In this study, we first verified the validity of the model by the decreased GFR at day 7 (Figure 1B), increased plasma creatinine and BUN (Supporting Information Table S2), the increased urinary albumin/creatinine ratio (uACR) in the anti‐GBM groups (Figure 1C), and by the tubular protein precipitates (casts) on renal histology resulting from proteinuria (Figure 1D).

Next, we determined the physiological effects of the six administrations of recombinant FGF23 or vehicle. To this end, we determined the concentrations of circulating FGF23. FGF23 was elevated 24 h after the last injection in both healthy and diseased FGF23 groups (Supporting Information Table S2). Further, the FGF23 administrations tended to decrease plasma phosphate compared to vehicle in healthy mice (Figure 1E). The fractional excretion of phosphate (FE_Pi_) was increased by FGF23 compared to vehicle, with an approximately threefold increase in healthy mice and a twofold increase in anti‐GBM mice (Figure 1E). However, FGF23 did not directly further aggravate renal function in the anti‐GBM disease model, as GFR was comparable at day 7 between the anti‐GBM groups treated with FGF23 or vehicle (*p* > 0.05; Figure 1C,D). Overall, the present data demonstrated substantial kidney disease in the anti‐GBM model, and evidence of a partial renal resistance to the phosphaturic actions of FGF23 in anti‐GBM disease.

### 3.2 FGF23 excess triggers proinflammatory renal responses and increased immune cell abundance in the diseased kidney

**FIGURE 2:**
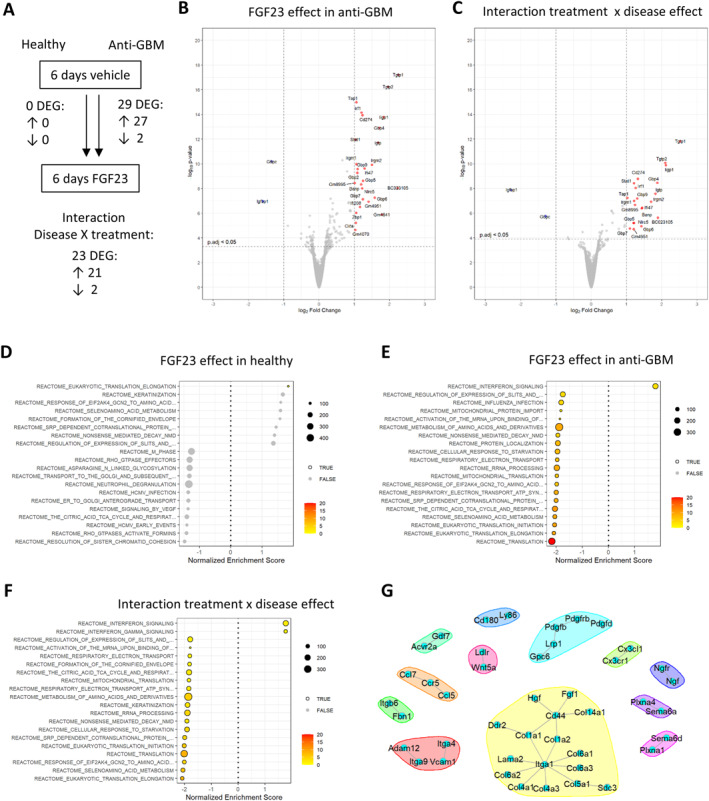
Six‐day course of FGF23 treatment induces renal transcriptional signatures of inflammatory responses and injury. (A) Indicates the number of differentially expressed genes according to experimental comparison in renal bulk RNA‐Seq. (B, C) Depict volcano plots of DEGs above a cutoff of adjusted *p* < 0.05 and log2‐fold change >1, in the comparison of FGF23 versus vehicle effect in mice with anti‐GBM (B) and the interaction between treatment and disease effect (C). (D–F) Depict significant Reactome gene set enrichment analyses of FGF23 effects in healthy mice (D), mice with anti‐GBM disease (E), and the interaction between treatment and disease effect (F). (G) Depicts a network of ligand–receptor interaction pairs that were significant for FGF23 versus vehicle comparisons in mice with anti‐GBM disease by bulk RNA‐Seq. The ligand–receptor interactions were inferred using R/BulkSignalR. Anti‐GBM, anti‐glomerular basement membrane disease. *N* = 3 for anti‐GBM groups and *n* = 4 for healthy groups.

FGF23 excess caused by recombinant hormone administration^22^, ^34^ or by *Fgf23* overexpression^27^ induces renal cell signaling via FGF receptors 1 and 3 and mitogen‐activated protein kinase phosphorylation, leading to gene expression of *Cyp24a1* encoding a vitamin D metabolizing enzyme. In mice with glomerular disease, however, FGF23 induced the renal injury marker *Lcn2*.^34^ To globally profile the transcriptional changes, we performed unbiased bulk RNA‐Seq in healthy mice and in mice with anti‐GBM disease treated with FGF23 or vehicle for 6 days. Overall, we found no differentially expressed genes 24 h after the last FGF23 injection in healthy mice, but 27 genes were upregulated and 2 genes were downregulated by FGF23 in anti‐GBM disease (Figure 2A, Supporting Information Table S3). Of note, the expression of the renal FGF23 co‐receptor *Klotho* in the anti‐GBM groups remained at 70% compared to the healthy groups (Supporting Information Table S3). In an interaction analysis, we next determined how FGF23 effects differ between anti‐GBM disease and healthy mice, revealing 23 DEGs (Figure 2A, Supporting Information Table S3). Figure 2B,C show the transcripts with a difference in FGF23 effect between anti‐GBM and healthy mice, including mRNA of *Tgtp1* and *Tgtp2* encoding T‐cell‐specific GTPases and *Irf1* encoding an interferon regulatory factor. Next, we performed a GSEA to gain insight into the overall pattern of affected transcripts. GSEA revealed proinflammatory FGF23‐dependent transcriptional signatures in anti‐GBM versus healthy mice, including an enrichment of interferon signaling (Figure 2D–F, Supporting Information Figure S1A–C).

Next, we aimed to better understand the mechanisms and involved signaling molecules by which FGF23 induced the observed proinflammatory renal transcriptional signatures. To this end, we performed ligand–receptor interaction analysis of the bulk RNA‐Seq data. This analysis revealed that the FGF23 effect in the anti‐GBM model included, on the one hand, signaling by several chemokines of the CC, CXC, and CX3C classes and their corresponding receptors (Figure 2G, Supporting Information Figure S2D). On the other hand, the ligand–receptor interaction analysis provided evidence of FGF23‐induced cell signaling by collagen‐binding integrins, Toll‐like receptor signaling, and TGF‐beta signaling, and the involved molecules *Vcam1*, *Pdgfrb*, and *Fbn* are all markers of injury and fibrogenesis (Figure 2G, Supporting Information Figure S2D,E). In summary, in‐depth bulk transcriptome analyses provided several layers of evidence pointing toward proinflammatory renal actions of FGF23 in the anti‐GBM disease model.

**FIGURE 3:**
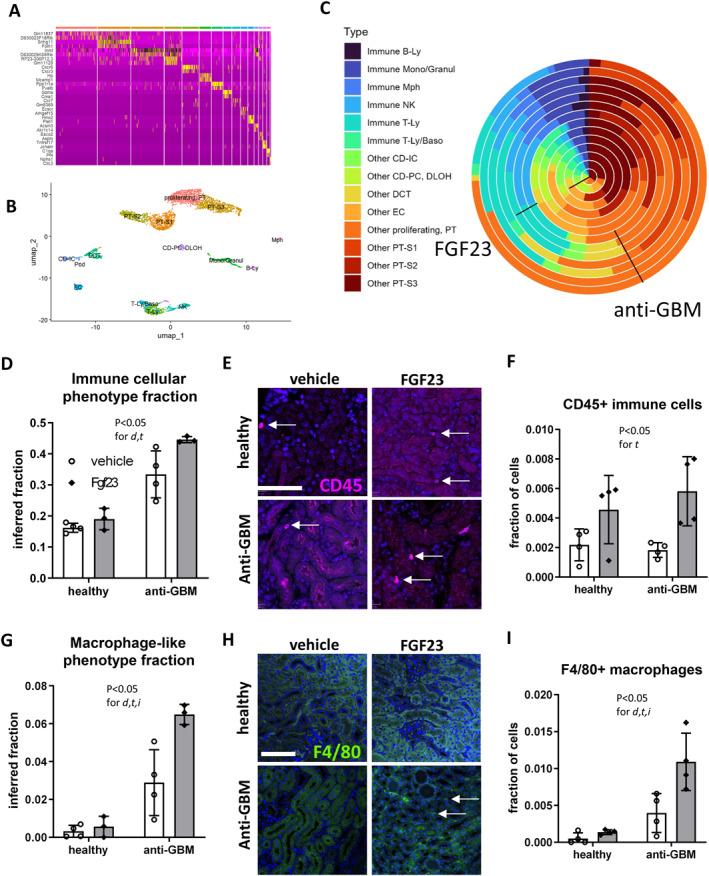
Bulk RNA‐Seq deconvolution and immunofluorescence staining reveal an FGF23‐driven increase in overall immune cell and macrophage abundance in the kidneys of mice with anti‐GBM disease. (A, B) Depict the annotation of renal cell clusters in the reanalysis of the single‐cell RNA‐Seq dataset GSE107585 of murine kidney from 7 sex‐mixed healthy C57BL/6 mice; see also Supporting Information Figure S2. (C) Shows a wedding pie plot of the bulk deconvolution of the renal cellular composition according to FGF23 treatment and anti‐GBM disease state, as indicated by labels. Overall renal immune cells and macrophage‐like cells are displayed by bulk deconvolution (D, G) and by immunofluorescence with automated quantification for CD45 (E, F) and F4/80 (H, I). RNA‐Seq: *N* = 3 for anti‐GBM groups and *n* = 4 for healthy groups. Immunofluorescence: *n* = 4 per group. Statistical analysis: two‐way ANOVA. anti‐GBM, anti‐glomerular basement membrane disease; Baso, basophil; CD, collecting duct; d, disease state; DCT, distal convoluted tubule; DLOH, descending limb of Henle; EC, endothelial cell; Granul, granulocyte; i, interaction; IC, intercalated cells; Ly, lymphocyte; Mono, monocyte; Mph, macrophage; NK, natural killer cell; PC, principal cells; PT, proximal tubule; S, segment; t, treatment.

Based on the above findings, we next examined the extent to which FGF23 may affect proinflammatory cytokines at the protein level. Therefore, we profiled 40 circulating immune proteins by a protein array in sera of healthy and anti‐GBM mice treated with vehicle or FGF23 (Supporting Information Figure S3). In healthy mice, FGF23 increased the circulating concentrations of soluble tumor necrosis factor receptor 1 (sTNFR1) (Figure 3A). By contrast, in mice with anti‐GBM disease, we found no FGF23‐induced alterations in immune proteins (Figure 3B). Consequently, a two‐factorial analysis of the interaction between treatment and disease state revealed sTNFR1 as the sole significantly altered immune protein (Figure 3C). Further, when comparing all animals regardless of FGF23 treatment, sTNFR2 was increased in anti‐GBM compared to healthy mice (Figure 3D). The individual results of sTNFR1 and sTNFR2 in each experimental group are shown in Figure 3E,F, and all comparisons are shown in Supporting Information Table S4. Overall, anti‐GBM disease in mice already induced a proinflammatory milieu in serum with elevated sTNFR1 and sTNFR2 concentrations, similarly as in human CKD.^35^, ^36^ Consequently, FGF23 had an effect on sTNFR1 only in disease‐free mice but did not alter the already elevated circulating sTNFR1 in mice with anti‐GBM disease.

**FIGURE 4:**
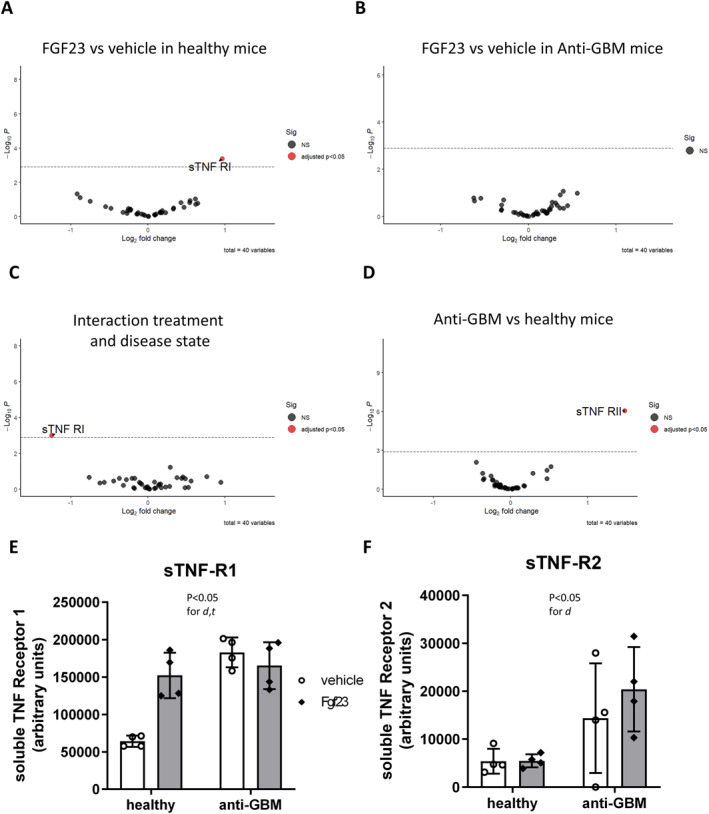
Immune protein profiling highlights an increase in circulating soluble tumor necrosis factor receptors induced by FGF23 and anti‐GBM disease in mice. A plasma cytokine protein array shows FGF23 effects in healthy male C57BL/6 mice (A) and in mice treated with nephrotoxic serum to induce anti‐GBM disease (B). The interaction between treatment and disease state (C) and the overall disease effect (D) are shown. (E–F) Depict analyses of soluble TNF receptors 1 and 2 by two‐way ANOVA. d, disease state; t, treatment. *N* = 4 biologically independent replicates per group. Anti‐GBM, anti‐glomerular basement membrane.

In view of the renal transcriptional signatures and cytokine data suggesting that FGF23 promotes a proinflammatory environment, we aimed to determine whether FGF23 caused an influx of immune cells to the kidney. Thus, we determined the relative abundance of different renal cell compartments by bulk transcriptome deconvolution and by immunofluorescence (Figure 4). For bulk deconvolution, we first reanalyzed and annotated existing single‐cell RNA‐Seq data of kidney from healthy C57BL/6 mice (Figure 4A, Supporting Information Figure S4). Second, we inferred the fraction of major cell types, including total immune cells and, specifically macrophages, from renal bulk transcriptomes of healthy and anti‐GBM mice treated with vehicle or FGF23 for 6 days (Figure 4C). FGF23 excess provided an additional increase in total immune cells (Figure 4D) and, specifically macrophages (Figure 4D), in addition to an observed increase in immune cells induced by anti‐GBM disease. Next, we validated these transcriptome‐derived findings using immunofluorescence staining of the kidney, confirming an increase in overall CD45‐positive immune cells (Figure 4E,F) and F4/80‐positive macrophages (Figure 4G,H) in FGF23‐treated mice compared to vehicle‐treated mice with anti‐GBM disease. Together, these findings provide strong evidence of an FGF23‐driven increase in immune cell content in the diseased kidney.

### 3.3 Time‐dependent and renal‐intrinsic proinflammatory effects of FGF23

**FIGURE 5:**
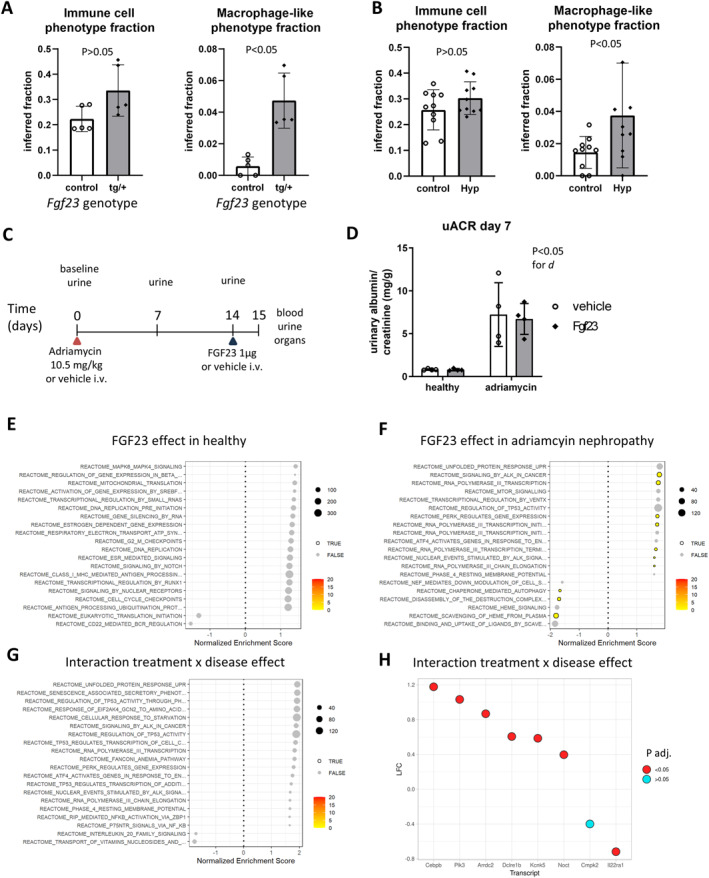
Renal immune cell recruitment driven by FGF23 excess is exposure time dependent. Renal microarray transcriptome datasets GDS3361 of male *Fgf23* transgenic and control mice or sex‐matched Hyp mice of dataset GDS879 (B) underwent bulk deconvolution with reference to single‐cell RNA‐Seq dataset GSE107585 of murine kidney from 7 sex‐mixed healthy C57BL/6 mice and, subsequently, visualization of overall fractions of inferred immune cells and macrophage‐like cells, as indicated. (C) Shows the experimental workflow of experiments with female BALB/c mice undergoing Adriamycin (doxorubicin) nephropathy followed by a single intravenous (IV) injection of FGF23 or vehicle. (D) Shows the urinary albumin/creatinine ratio 7 days after induction of Adriamycin nephropathy. Statistical analysis: two‐way ANOVA. d, disease state. (E–G) Show significant Reactome gene set enrichment analyses of renal FGF23 effects in healthy mice (E), mice with Adriamycin nephropathy (F), and the interaction between treatment and disease effect (G). (H) Depicts the log‐fold change of 8 transcripts with lowest adjusted *p*‐value in the interaction analysis of FGF23 effect in diseased versus FGF23 effect in healthy mice in a 2 × 2 factorial design. *N* = 5 (A), 10 (B), or 4 (C–H) biologically independent replicates per group.

With evidence of excessive FGF23 causing proinflammatory effects in the diseased kidney, we aimed to determine the time dependency of this aberrant FGF23 effect. First, we used bulk transcriptome deconvolution of existing microarray data from kidneys of mice transgenically overexpressing Fgf23 and mice harboring a mutation in the *Fgf23* repressor gene *Phex* (Hyp mouse) and their respective controls as models of long‐term FGF23 excess. This analysis revealed that genetically induced *Fgf23* excess tends to increase the total renal immune content (Figure 5A) and significantly increases the inferred renal macrophage content (Figure 5B). Conversely, we also studied mice with a short‐term treatment of FGF23 in a different disease model of Adriamycin nephropathy (Figure 5C): We first induced glomerular and renal tubular disease using Adriamycin in female mice, leading to an over eightfold increase in urinary albumin/creatinine ratio (ACR) (Figure 5D). In addition, serum BUN tended to be higher, and serum creatinine was significantly increased in mice with Adriamycin‐induced nephropathy (Supporting Information Table S2). We then administered a single intravenous injection of recombinant FGF23 24 h prior to euthanasia in both healthy mice and mice with Adriamycin‐induced nephropathy. There was no change in circulating FGF23, phosphatemia, or urinary phosphate excretion at the 24‐h interval (Supporting Information Table S2). In renal RNA‐Seq analysis of these mice (Figure 5F–H, Supporting Information Table S5), the top DEGs in an interaction analysis between FGF23 treatment and disease state included the proinflammatory transcription factor *Cebpb* and an increased DNA damage transcript, *Dclre1b* (Figure 5H, Supporting Information Table S5). GSEA pointed toward FGF23 decreasing chaperone‐mediated autophagy in the diseased kidney, with substantially fewer overall effects induced by a single FGF23 injection at the 24‐h interval (Figure 5F–G). Overall, a long‐term genetic induction of FGF23 excess increased renal macrophage content in otherwise healthy mice, whereas a short‐term FGF23 treatment of 24 h induced only limited potential proinflammatory FGF23 effects in mice with kidney disease and none in healthy mice.

**FIGURE 6:**
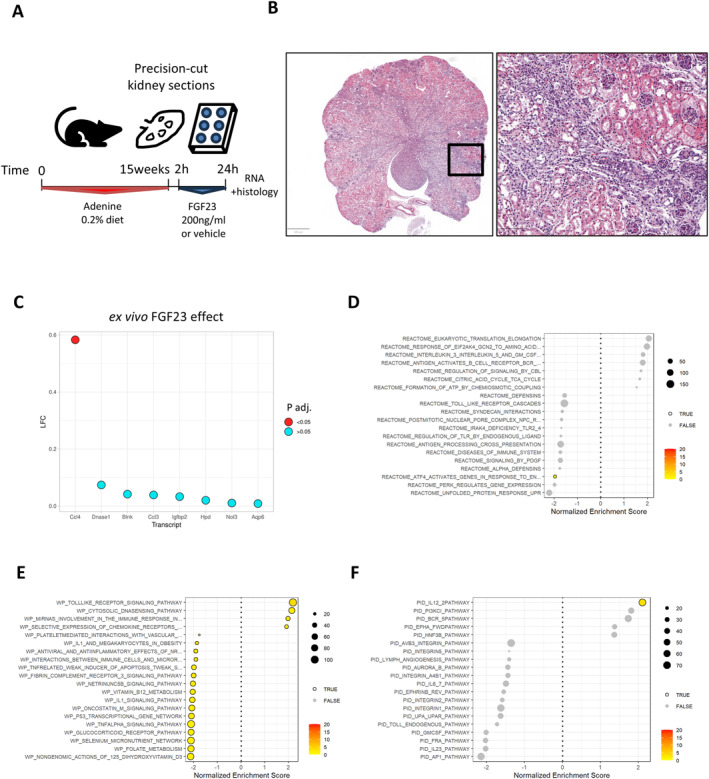
Intrarenal proinflammatory effects of FGF23 applied ex vivo in PCKS. (A) Male DBA/2J mice underwent dietary treatment with 0.2% adenine for 15 weeks, followed by organ collection and preparation of 300 µm PCKS for a 24‐h treatment with FGF23 or vehicle ex vivo. No disease‐free controls were used for this substudy. (B) Depicts the fibrotic changes in a representative 4 µm section of PCKS stained with hematoxylin and eosin (left scale bar, 500 µm; right, 100 µm). (C) Shows the log‐fold change of upregulated transcripts with the lowest adjusted *p*‐value in the FGF23 versus vehicle comparison. (D–F) Show gene set enrichment analyses of FGF23 effects in Reactome (D), WikiPathways (E), and Pathway Interaction Database (F) gene sets. *N* = 4 biologically independent replicates per group. PCKS, precision‐cut kidney slices.

Systemic proinflammatory effects of FGF23 have been reported to be mediated via the liver, where FGF23 drives cytokine expression via FGFR4.^8^ However, we hypothesized that renal tissue‐resident cells could also contribute to proinflammatory actions of FGF23. Therefore, we employed an isolated ex vivo model system and studied FGF23 signaling in PCKS (Figure 6A) from mice subjected to a classic model of adenine‐induced nephropathy with advanced tubulointerstitial fibrosis (Figure 6B) and elevated concentrations of circulating intact and C‐terminal FGF23 (Supporting Information Table S2). In an RNA‐Seq analysis of PCKS from these mice, a 24‐h treatment with FGF23 compared to vehicle resulted in 1 transcript with significantly increased expression, namely, *Ccl4*, encoding a proinflammatory macrophage/fibroblast chemokine (Figure 6C, Supporting Information Table S6). GSEA of the Reactome database revealed no significant FGF23‐induced gene set alterations in PCKS (Figure 6D), but GSEA by WikiPathways showed enrichment of Toll‐like receptor signaling (Figure 6E), and the Pathway Interaction Database showed enrichment of the interleukin‐12 pathway (Figure 6F), a proinflammatory early cytokine that leads to secondary release of interferon‐gamma by natural killer and T cells.^37^ Overall, these data demonstrate evidence of kidney‐intrinsic proinflammatory responses to FGF23 in an ex vivo PCKS model of diseased mouse kidney.

### 3.4 Circulating FGF23 concentrations are associated with renal immune cell infiltration in human IgA nephropathy

**FIGURE 7:**
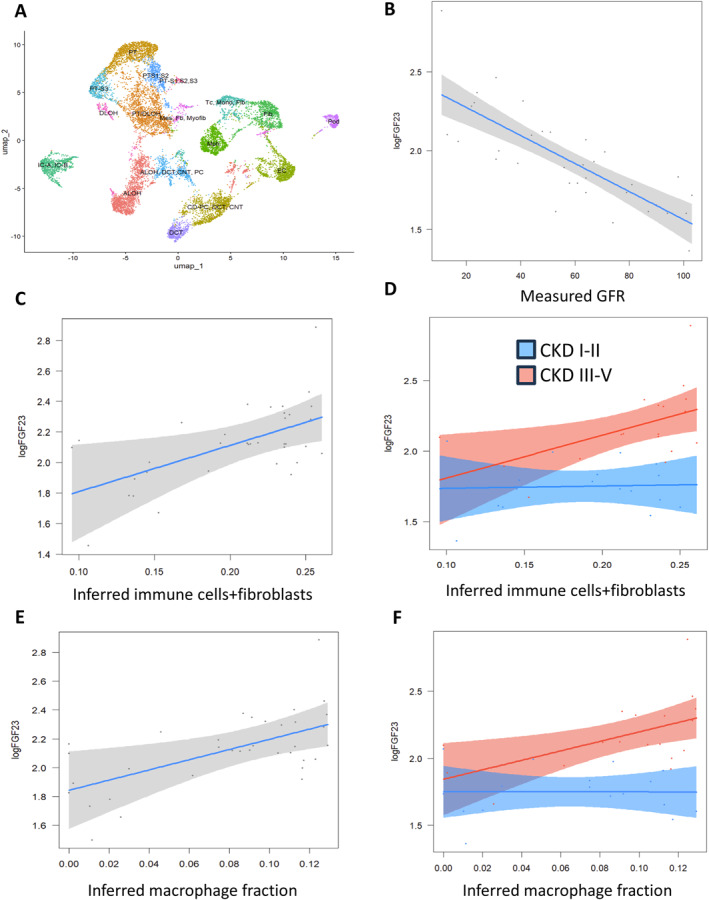
FGF23 is associated with renal immune cell content in human patients with IgA nephropathy. As a reference, single‐nucleus RNA‐Seq data from 5 human kidney biopsies (GSE199711) of 2 healthy controls and 3 patients with chronic kidney disease (CKD) were reanalyzed and annotated (A); see also Supporting Information Figure S5. 35 patients with IgA nephropathy from the Karolinska Kidney Biopsy Cohort showed an inverse univariable association between circulating FGF23 and measured glomerular filtration rate (GFR) (B). The associations between transcriptome‐inferred renal fibroblasts and immune cells (C–D) or macrophages (E–F) and circulating FGF23 are shown, with adjustment for GFR, 25OH‐vitamin D and parathyroid hormone. (D, F) Show disaggregated data stratified by GFR in CKD stages I–II and III–V.

Finally, we aimed to determine whether the link between elevated FGF23 and renal immune cell content found in mice can be translated to human kidney disease. To this end, we reassessed a subcohort of 59 patients with IgA nephropathy, a glomerular disease with varying degrees of renal inflammation and dysfunction whose kidney biopsies had undergone routine histopathological examination by board‐certified pathologists and transcriptome profiling of the nonglomerular (tubular) kidney fraction by RNA‐Seq. Patient characteristics are described in Supporting Information Table S7. We first reanalyzed and annotated a single‐nucleus RNA‐Seq dataset as a reference (Figure 7A, Supporting Information Figure S5) to infer renal immune cell content. As a validation step, we verified that the transcriptome‐inferred immune content of an immune cell/fibroblast cluster and of a macrophage cluster were associated with histopathological immune cell infiltration (Supporting Information Table S8). We also verified the inverse association between FGF23 and renal function (Figure 7B). We then tested the association between transcriptome‐inferred immune cell content and circulating FGF23. There were associations between transcriptome‐inferred immune cell content and FGF23, predominantly in those with moderate to advanced renal dysfunction (Figure 7C–F). In addition, we found associations between circulating FGF23 and several histopathological parameters of glomerular injury and tubulointerstitial immune infiltration in univariable or adjusted analyses (Supporting Information Table S9). Overall, the present data provide evidence of a link between FGF23 excess and immune cell infiltration in IgA nephropathy.

## 4 DISCUSSION

In this study, we set out to describe the pathophysiological effects of FGF23 signaling in kidney disease, with the intention to detect quantitative and qualitative aberrations in FGF23 signaling. In healthy mice, we detected no transcriptional alterations in the kidney at the 24‐h interval after the last treatment with FGF23 compared to vehicle. However, mice with anti‐GBM disease displayed proinflammatory cytokine expression and an excessive renal abundance and/or activity of macrophages and overall immune cells after the six‐day regimen of FGF23 compared to vehicle. An in‐depth transcriptomic analysis of ligand–receptor interactions in the kidney revealed FGF23‐driven overactive collagen‐integrin signaling that is associated with inflammation^38^ and additional signaling involving mediators of tissue damage and acute kidney injury (AKI)‐to‐CKD transition, such as Pdgfrb and Vcam1.^39^ Over the long term, a genetically induced excess of FGF23 in two transgenic mouse strains increased renal macrophage content even in the absence of kidney disease. Finally, even ex vivo experiments in PCKS of kidneys isolated from mice with adenine nephropathy revealed increased expression of *Ccl4*, encoding a proinflammatory macrophage/fibroblast chemokine associated with cardiovascular events and disease,^40^ and transcriptional signatures of inflammatory signaling. These findings were preserved in associations between circulating FGF23 and renal immune cell content in human patients with advanced kidney disease.

**FIGURE 8:**
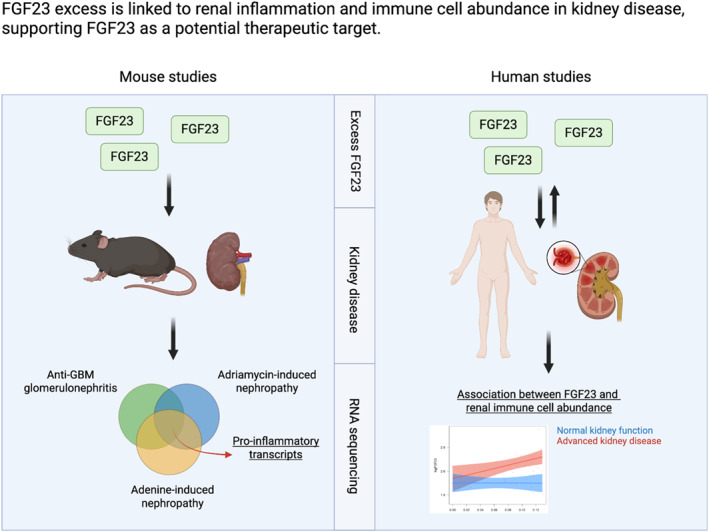
FGF23 excess is linked to renal inflammation and immune cell abundance in kidney disease, supporting FGF23 as a potential therapeutic target. A graphical model made in BioRender integrates the obtained data and illustrates that FGF23 excess in murine kidney disease models causes a proinflammatory environment (left panel). In human data, an association exists between systemic FGF23 concentrations and renal immune cell abundance in advanced kidney disease (right panel).

Overall, the present data point toward a pathogenic potential of FGF23 excess over a long‐term duration or in kidney disease in a variety of settings, including patients, three experimental mouse strains, different sexes, and FGF23 administration routes and dosing (Figure 8). Singh et al. have previously reported that FGF23 induces FGFR4‐mediated calcineurin signaling in the liver that then contributes to hepatic C‐reactive protein expression and systemic inflammation.^8^ However, using the present approach of PCKS, our obtained results suggest that FGF23 may have direct kidney‐specific adverse consequences in diseased kidney tissue, even in an experimental setup of PCKS bypassing the proinflammatory systemic effects of FGF23 in the liver. Further, we previously reported rapid oxidative changes occurring in kidney tissue as a result of FGF23 administration,^22^ which may additionally promote a proinflammatory environment when occurring in kidney disease, a disease known to cause profound oxidative stress at the tissue level.^41^ Finally, our findings demonstrate a time dependency of FGF23 excess, with no significant global proinflammatory GSEA findings in short‐term FGF23 treatment in mice with or without kidney disease. However, chronic FGF23 excess caused transcriptional evidence of renal macrophage overabundance even in mice without kidney disease. This correlates well with the pathophysiology observed in patients with AKI, where initial FGF23 excess was predictive of future transition to CKD.^42^

A large body of evidence demonstrates that FGF23 excess is associated with adverse cardiovascular outcomes and mortality.^15^, ^43^, ^44^ Mechanistically, recent data by our group also point toward an adverse proinflammatory effect of FGF23 in experimental atherosclerosis.^10^ As a main implication of this study, our findings support the idea that it may be beneficial to reduce the amount of circulating FGF23 excess in kidney disease in order to prevent its toxicity. However, as complete FGF23 neutralization increased phosphate toxicity and mortality in rats with CKD,^13^ a more sophisticated and nuanced approach is warranted: Potential strategies can include a partial inhibition of FGF23 action using inhibitory C‐terminal FGF23 peptides that improved uremic cardiomyopathy^6^ or anemia,^45^ or more traditional strategies to tackle the known drivers of FGF23 excess. One of the mainstays of CKD‐MBD therapy is the reduction of dietary intake and enteral absorption of phosphate, which improved anemia and sarcopenia in experimental CKD^7^ and diminished metabolic acidosis and mortality in experimental AKI.^46^ In human patients with CKD, elevated serum phosphate is associated with an increased risk of death and cardiovascular disease.^47^ Simply recommending to patients with advanced CKD to reduce their phosphate intake did not improve mortality.^48^ Pharmacological restriction of phosphate uptake induced by phosphate‐binding agents is associated with lower overall mortality in end‐stage kidney disease,^49^ whereas in predialysis populations, the effect of phosphate binders on patient outcomes remains, so far, inconclusive.^50^ As hyperphosphatemia is one of the key drivers of FGF23 excess in CKD, it is crucial to address phosphate intake in the context of FGF23 toxicity but also to consider other drivers (and consequences) of FGF23 excess, such as chronic inflammation, to ultimately improve clinical outcomes in CKD‐MBD.

Strengths of this study include the variation in animal strains, sexes, and FGF23 application, in addition to the in‐depth histological, transcriptomic, and cytokine subproteome profiling in combination with advanced bioinformatics analyses. We chose relevant disease contexts in both murine and human data, and we cross‐validated key findings by immunofluorescence and histopathology. However, this study also has some limitations. First, in wet‐laboratory work, we used human FGF23 to be able to directly quantify the injected recombinant protein by ELISA. This protein has its expected physiological effects in mice, and although human FGF23 is used in many mouse studies,^27^, ^51^, ^52^, ^53^ human FGF23 might theoretically induce an allogenic immune response. However, an allogenic immune response would be expected to occur in all FGF23‐treated experimental groups, which was not the case. Additionally, we validated our findings also in renal transcriptomes of Hyp mice that have excessive levels of endogenous murine FGF23.^28^ Next, we observed a discrepancy between transcriptome‐inferred and immunofluorescence‐estimated cell fractions. This may be, in part, attributable to the fact that our bulk deconvolution procedure was performed using healthy mouse reference single‐cell RNA‐Seq data, and therefore some adaptive or injured epithelial cells of the bulk transcriptomes could have been misclassified as immune cells based on their transcriptional profile resembling immune cells. However, using standard markers CD45 and F4/80 in immunofluorescence, we confirmed the main trend of increased immune cells and macrophages induced by FGF23 excess in the anti‐GBM model. Transcriptomic results of the other disease models were not validated further. In addition, the PCKS study was conducted only in diseased animals, which limits its interpretation. Next, the human dataset is of a relatively small sample size and included a one‐time measurement of FGF23 only. Finally, in this study, we almost exclusively focused on the kidney, not engaging with the proinflammatory effects of the liver under FGF23 excess,^8^ the potential effects of phosphate excess on gut microbiota that can additionally generate bacterial‐derived uremic toxins that may promote cardiovascular morbidity,^54^ and we did not consider the known direct renal tubular phosphate toxicity, which FGF23 excess may promote by increasing phosphate concentrations in the renal tubular lumen.^44^

As a consequence of the obtained data, there is an incentive for future studies to investigate potential protective effects for uremic complications by carefully diminishing inflammatory FGF23 signaling both in CKD and also in the context of AKI to prevent the AKI‐to‐CKD transition.

To conclude, we employed several complementary approaches to determine adverse renal cell signaling induced by FGF23 in kidney disease, and we uncovered substantial kidney‐intrinsic proinflammatory effects induced by FGF23 and thereby further highlight FGF23 excess as a potential therapeutic target in CKD‐MBD.

## AUTHOR CONTRIBUTIONS

Matthias B. Moor and Hannes Olauson conceived the study. Mikhail Burmakin, Hannes Olauson, Anna Witasp, Annika Wernerson and Matthias B. Moor participated in experimental design. Mikhail Burmakin, Anna Levin, Gül Gizem Korkut and Matthias B. Moor performed experiments. Annette Bruchfeld, Anna Levin, Anna Witasp, Peter Bárány and Annika Wernerson obtained clinical data. Matthias B. Moor and David Brodin performed computational analyses. All authors participated in data interpretation. Hannes Olauson and Jaakko Patrakka provided crucial laboratory materials. Hannes Olauson, Jaakko Patrakka and Matthias B. Moor acquired funding for the research. Hannes Olauson and Matthias B. Moor supervised the research. Matthias B. Moor wrote the manuscript. All authors critically read and commented on the manuscript and agreed to manuscript submission.

## CONFLICT OF INTEREST STATEMENT

Jaakko Patrakka's research laboratory was financially supported by AstraZeneca and Guard Therapeutics International. Mikhail Burmakin was financially supported by Guard Therapeutics International. Annette Bruchfeld received consultancy fees from Amgen, AstraZeneca, Boehringer Ingelheim, CSL Vifor, Otsuka, and Sobi and payment or honoraria for lectures, presentations, speaker's bureaus, manuscript writing, or educational events from AstraZeneca, Bayer, Boehringer Ingelheim, ChemoCentryx, CSL Vifor, Fresenius, GlaxoSmithKline, and Otsuka. All other authors declare no competing interests.

## ETHICS STATEMENT

All animal experiments were carried out at Karolinska Institutet following the protocols approved by the Ethical Committee on Research Animal Care (Linköping Animal Experimentation Ethics Committee; DNR 1336). The Karolinska Kidney Biopsy Project was approved by the Swedish Ethical Review Authority.

## ACCESSION CODES

The murine bulk RNA‐Seq datasets generated within this study are accessible at Gene Expression Omnibus under the accession number GSE272289. Human patients' bulk RNA‐Seq data have previously been deposited under the accession number GSE199711. Further datasets used in this study were GSE107585, GSE199711, GDS3361, and GDS879, as described in the Materials and Methods section. Data from the Karolinska Kidney Biopsy Cohort can be requested from our Research Data Office (rdo@ki.se) at Karolinska Institutet.

## Supporting information

Supporting Information S1

Table S1

Table S2

Table S3

Table S4

Table S5

Table S6

Table S7

Table S8

Table S9

## Data Availability

The bulk RNA‐Seq datasets generated within this study are accessible at Gene Expression Omnibus under the accession number GSE272289. Existing single‐cell RNA‐Seq data were retrieved from Gene Expression Omnibus by the accession numbers GSE107585 and GSE199711. Existing renal microarray transcriptome datasets GDS3361 and GDS879 were retrieved from the GEO dataset browser. Analyses and visualizations were performed using adaptations of existing code from the tools BulkSignalR, Seurat, scCustomize, bisque, EnhancedVolcano, and SuperPlotsOfData. Data from the Karolinska Kidney Biopsy Cohort cannot be deposited to public repositories due to ethical restrictions but are available upon request; requests for access to the data can be put to our Research Data Office (rdo@ki.se) at Karolinska Institutet.
